# *TNFA* Haplotype Genetic Testing Improves HLA in Estimating the Risk of Celiac Disease in Children

**DOI:** 10.1371/journal.pone.0123244

**Published:** 2015-04-27

**Authors:** Elisa Rossi, Daniela Basso, Carlo-Federico Zambon, Filippo Navaglia, Eliana Greco, Michela Pelloso, Serena Artuso, Andrea Padoan, Matilde Pescarin, Ada Aita, Dania Bozzato, Stefania Moz, Mara Cananzi, Graziella Guariso, Mario Plebani

**Affiliations:** 1 Department of Medicine—DIMED, University of Padova, Padova, Italy; 2 Department of Laboratory Medicine, University—Hospital of Padova, Padova, Italy; 3 Unit of Pediatric Gastroenterology, Department of Women and Children's Health, University-Hospital of Padova, Padova, Italy; Hospital Israelita Albert Einstein, BRAZIL

## Abstract

**Background:**

TNF-α and IFN-γ play a role in the development of mucosal damage in celiac disease (CD). Polymorphisms of *TNFA* and *IFNG* genes, as well as of the *TNFRSF1A* gene, encoding the TNF-α receptor 1, might underlie different inter-individual disease susceptibility over a common HLA risk background. The aims of this study were to ascertain whether five SNPs in the *TNFA* promoter (-1031T>C,-857C>T,-376G>A,-308G>A,-238G>A), sequence variants of the *TNFRSF1A* gene and *IFNG* +874A>T polymorphism are associated with CD in a HLA independent manner.

**Methods:**

511 children (244 CD, 267 controls) were genotyped for HLA, *TNFA* and *INFG* (Real Time PCR). *TNFRSF1A* variants were studied (DHPLC and sequence).

**Results:**

Only the rare *TNFA*-1031C (OR=0.65, 95% CI:0.44-0.95), -857T (OR=0.42, 95% CI:0.27-0.65), -376A (OR=2.25, 95% CI:1.12-4.51) and -308A (OR=4.76, 95% CI:3.12-7.26) alleles were significantly associated with CD. One *TNFRSF1A* variant was identified (c.625+10A>G, rs1800693), but not associated with CD. The CD-correlated *TNFA* SNPs resulted in six haplotypes. Two haplotypes were control-associated (CCGG and TTGG) and three were CD-associated (CCAG, TCGA and CCGA). The seventeen inferred haplotype combinations were grouped (A to E) based on their frequencies among CD. Binary logistic regression analysis documented a strong association between CD and HLA (OR for intermediate risk haplotypes=178; 95% CI:24-1317; OR for high risk haplotypes=2752; 95% CI:287-26387), but also an HLA-independent correlation between CD and *TNFA* haplotype combination groups. The CD risk for patients carrying an intermediate risk HLA haplotype could be sub-stratified by *TNFA* haplotype combinations.

**Conclusion:**

*TNFA* promoter haplotypes associate with CD independently from HLA. We suggest that their evaluation might enhance the accuracy in estimating the CD genetic risk.

## Introduction

Celiac disease (CD), an autoimmune enteropathy, develops in genetically predisposed individuals after the exposure to gluten-derived peptides [1,2]. CD patients make antibodies specific for gluten peptides, but also for autoantigens, tissue transglutaminase (tTG) being a highly specific autoantigen, and develop enterocytes destruction by CD8^+^ T cells [2]. Moreover, numerous innate and adaptive immune response alterations occur in both the intestinal epithelial layer and the lamina propria, resulting in the infiltration of leukocytes, the activation of dendritic cells and CD4^+^ T cells with an increased expression, and the release of IL-15, interferon (IFN)-γ and possibly IL-21 [3]. In the mucosa of CD patients, macrophages are also activated, these cells being the main producers of the tumor necrosis factor (TNF)-α, another relevant cytokine in CD pathogenesis [4–7].

The maintenance of the immune alterations seen in CD intestinal mucosa is strictly dependent on gluten ingestion, which was demonstrated to increase TNF-α, not IFN-γ expression in CD patients, and to increase IFN-γ, not TNF-α in “non-celiac gluten sensitivity” patients [7]. Gluten derived peptides, which undergo enzymatic deamidation by tTG, are transformed into deamidated gluten derived peptides, which are preferentially recognized by lamina propria CD4^+^ T cells in the presence of disease-associated HLA molecules [2]. The HLA locus, the most relevant genetic factor for CD, accounts for 40 to 50% of the genetic variance occurring in patients with the disease. The HLA-DQ2 haplotype (DQA1*05/DQB1*02, also known as DQ2.5) is expressed in 90% of CD cases, but also in one third of the general population, while HLA-DQ8 haplotype (DQA1*03/DQB1*0302) or another variant of HLA-DQ2 (DQA1*02/DQB1*02, also known as DQ2.2) is expressed in about 5%. The influence of HLA on CD susceptibility shows a dose effect. Individuals can be classified as being at a high or intermediate risk of CD on the basis of the HLA-DQ haplotype and on the number of DQB1*02 carrying alleles [8–11].

Several non-HLA genes, mainly derived from GWAS, have recently been identified as new susceptibility factors for CD. So far, 39 loci with 57 independent association signals have been described and overall they have been estimated to account for about 14% of the genetic variance of the disease [2]. Many of the newly discovered CD associated loci harbor genes that are related to the immune response, particularly B and T cell function [12–14] and some polymorphisms that have emerged from GWAS are indirectly related to the physiology of IFN-γ and/or TNF-α [13,15]. IFN-γ and TNF-α production might be dependent also on *IFNG* and *TNFA* genes polymorphisms, and in particular on *IFNG* +874A>T and *TNFA* -308G>A which have a functional significance [16–18]. Few and contrastive studies are available in literature focusing on *IFNG* +874A>T polymorphism in CD [19,20], while some studies support the existence of an association between CD and the *TNFA* -308 A allele [21–25]. Notably, it has been observed that this latter association is independent of the HLA-DQ2 haplotype despite the linkage between *TNFΑ* gene and HLA class II genes, both located in close proximity on the human chromosome 6p21.3 region [26]. Furthermore, although the *TNFA* -308 A allele is reported to be correlated with a higher transcription than the G allele [26], this finding might depend solely on the fact that the allele belongs to specific extended *TNFA* haplotypes [27].

TNF-α acts by binding to TNF-α receptors, mainly TNFΑ-R1 and TNFΑ-R2. Mutations in the TNFΑ-R1 extracellular domain are associated with the auto-inflammatory TNF receptor-associated periodic syndrome (TRAPS) but may also be involved in CD, which is included in the autoinflammatory-autoimmune continuum [28]. The hypothesis that autoinflammation may play a pathogenic role in CD is supported by the recent findings of Palova-Jelinkova et al. [29], who demonstrated that the breakdown of wheat gliadin by pepsin induces a robust IL-1β and IL-1α secretion by peripheral blood mononuclear cells through the activation of innate immune pathways, including the NLRP3 inflammasome. To our knowledge, no study in the literature has investigated whether sequence variations of the TNF-α receptor gene *TNFRSF1A* are associated with CD.

We hypothesized that a combined analysis of *TNFA* promoter haplotypes, *TNFRSF1A* sequence and of *IFNG* +874A>T polymorphism will allow to identify HLA-DQ2/DQ8 independent genetic risk factors for CD. To verify this hypothesis, we thought to study the *IFNG* +874A>T and five polymorphisms of the *TNFA* promoter (-1031T>C, -857C>T, -376G>A, -308G>A, -238G>A) and to inspect for variants of the *TNFRSF1A* sequence in a large retrospective cohort of CD and control children.

## Materials and Methods

### Ethics statement

The parents of all the children gave their fully informed written consent to the study for their offspring. The study was approved by the Local Institutional Ethic Committee (“Comitato Etico per la Sperimentazione, Azienda Ospedaliera di Padova”, ce.sperimentazione@sanita.padova.it).

### Patients

In the present retrospective cohort study, we investigated a total of 511 young patients coming from the North East of Italy (Table 1): 198 boys (mean and median age 9 [range 4–12] years) and 313 girls (mean and median age 9 [range 4–12]) years. All children consecutively underwent upper gastrointestinal endoscopy for persistent abdominal symptoms. In all cases, fasting blood for DNA and IgA tTG analyses (S1 Materials and Methods), duodenal and gastric biopsies for histology were taken. CD was histologically diagnosed in 244 (cases) and ruled out in the remaining 267 children (controls). The endoscopic findings and intestinal type lesions classified following theMarsh–Oberhuber criteria for celiac lesions [30], are detailed in Table 1.

**Table 1:** Patients’ details and clinicopathological characteristics.

	CD	Controls
**Patients, n**	244	267
**Females, n (%)** ^a^	167 (68%)	146 (55%)
**Mean age ±s.d. (years)** ^b^	7±4	10±4
**Age range (years)**	1–23	0.5–19
**Endoscopic findings:**		
**Normal**	19	167
**Esophagitis / Hiatus hernia**	0 / 0	17 / 6
**Antral hyperemia / Diffuse gastric hyperemia / Duodenitis**	3 / 0 / 1	24 / 11 / 1
**Nodularity of the antral mucosa / Antrum and corpus nodularity**	4 / 0	19 / 12
**Duodenal nodular mucosa with mosaic pattern / Duodenal nodular mucosa with mosaic pattern and scalloping**	149 / 68	9 / 1
**Histology (Marsh-Oberhuber classification):**		
**No Lesions**	0	262
**Type 1**	9	5
**Type 2**	16	0
**Type 3**	219 (3a = 34, 3b = 51, 3c = 134)	0
***H*. *pylori* infection** ^c^	12	50
**Mean tTG±s.d. (Units)** ^d^	153±79	5±9

CD, celiac disease

^a^ Chi-square test: 10.17, p = 0.001

^b^ Student’s t test for unpaired data: t = 7.13, p<0.0001

^c^ Chi-square test: 20.09, p<0.0001

^d^ Student’s t test for unpaired data: t = 30.14, p<0.0001

### *TNFA* and *IFNG* genotyping

Genomic DNA, extracted from EDTA-K_2_ peripheral blood by the Qiamp DNA blood maxi kit (QIAGEN S.p.A., Milan, Italy), was used to study *IFNG* +874A>T (rs2430561) and *TNFA* (-1031T>C, rs1799964; -857C>T, rs1799724; -376G>A, rs1800750; -308G>A, rs1800629; -238G>A, rs361525) polymorphisms, to detect *TNFRSF1A* variants and to genotype HLA. Single nucleotide polymorphisms of *IFNG* and *TNFA* genes were assayed by real-time PCR (ABI Prism 7900, Applied Biosystem, Foster City, CA, USA) as previously described in details [31,32].

### *TNFRSF1A* analysis

Exons 2, 3, 4 and 6 of the *TNFΑRSF1A* gene were studied using denaturing high-performance liquid chromatography (DHPLC; Wave 2100 Fragment Analysis, Transgenomic, Omaha, NE, USA) and gene sequencing (ABI PRISM 3130 Genetic Analyzer, Applied Biosystem) according to D’Osualdo et al. [33]. Details are reported in S1 File and S1 Table.

### HLA genotyping

A real-time polymerase chain reaction was employed by means of Taqman probes using an ABI Prism 7900 HT sequence detection system. Primers and probes for HLA-DQA1*0201, HLA-DQA1*03 and HLA-DQA1*05 detection were described by Fernandez et al. [34]. For HLA-DQB1*02 and HLA-DQB1*0302 alleles typing, primers were those described by Ferstlet al. [35]. Homozygous status was evaluated for the HLA-DQB1*02 allele by the set up of RT-PCR reactions to detect most of the already known *HLA-DQB1* alleles different from *02 [35]. Primers and probes were aligned with sequences reported in the IMGT/HLA database (http://www.ebi.ac.uk/ipd/imgt/hla/) and were purchased by Invitrogen Life Technologies (Monza, Italy). The analyses were made starting with about 100 ng DNA in a final volume of 25 μl containing 1X Taqman Universal PCR Master Mix (Applied Biosystems), 900 nM primers and 250 nM probes. The thermocycling conditions were 50°C for 2 minutes, 94°C for 7 minutes, followed by 42 cycles at 92°C for 20 seconds, 60°C for 1 minute and 20 seconds.

### Statistics

The statistical analysis of data was made by using the chi-square test, binary logistic regression analysis, Student’s t test for unpaired data, Shapiro-Wilk W test for normal data, one way Anova (Stata 13.1, StataCorp, Lakeway Drive, TX, USA). The Hardy-Weinberg equilibrium test and pairwise linkage disequilibrium calculation analysis were done using Arlequin, version 2.000 (http://cmpg.unibe.ch/software/arlequin3/). Haplotypes with frequency and odds ratios were estimated by the retrospective profile-likelihood approach (dominant model; haplologit package; Stata 13.1) [36].

## Results

In the retrospective cohort of 511 children and adolescents evaluated in this study, CD was diagnosed in 244 patients (cases) and ruled out in the remaining 267 (controls). Among controls mean age was higher, female gender lower and *H*. *pylori* infection more frequent than in CD (Table 1). Accordingly, age, gender and *H*. *pylori* infection were considered as potential confounding factors in all subsequent statistical analyses.

### Dose effect of *HLA-DQA1* and *HLA-DQB1* genetics on CD susceptibility

HLA-DQ haplotypes were classified in agreement with Megiorni et al. [10] on the basis of the combination between *HLA-DQA1* and of *HLA-DQB1* alleles. Table 2 shows the HLA-DQ haplotypes, the corresponding *HLA-DQA1* and *HLA-DQB1* alleles, the number of patients bearing each individual haplotype and its frequency in CD cases and controls. In Table 2, the Odds Ratio (OR) and the 95% Confidence Interval (95% CI) for any HLA-DQ haplotype, calculated with respect to HLA-DQ negative patients, are also reported together with the estimated post-test probability of CD, considering that its reported prevalence among the general population of children and adolescents from the same geographic area is 1:184 [37]. The HLA-DQ2.5 homozygous haplotype was associated with the highest risk of CD (1:7). Interestingly, the homozygous HLA-DQB1*02 allele alone increased CD risk to levels comparable with those of HLA-DQ2.5 heterozygotes, while HLA-DQ8 had only a modest effect on CD risk (1:450).

**Table 2:** HLA haplotype and CD risk.

HLA	*HLA-DQA1* alleles	*HLA-DQB1* alleles^a^	Total, Nr.	Cases, Nr.(frequency)	Controls, Nr.(frequency)	OR (95%CI)^b^	p-value	Adjusted p-value	Risk of CD^c^
**Neg**	Any	X/X	159	1 (0.01)	158 (0.99)	Ref.	-	-	1:18216
**DQ8**	*03/any	*0302/X	24	7 (0.29)	17 (0.71)	83 (9–742)	**<0.0001**	**<0.0001**	1:450
**B2, hetero**	*05 neg	*02/X	27	8 (0.30)	19 (0.70)	63 (7–547)	**<0.0001**	**<0.0001**	1:429
DQ8/B1*02 pos	*03/*05 neg	*0302/*02	8	3 (0.37)	5 (0.63)	104 (8–1375)	**<0.0001**	**<0.0001**	1:313
**DQ2.5 hetero**	*05/any	*02/X	162	104 (0.64)	58 (0.36)	284 (38–2107)	**<0.0001**	**<0.0001**	1:103
**DQ2/DQ8**	*03/*05	*0302/*02	14	10 (0.71)	4 (0.29)	585 (52–6523)	**<0.0001**	**<0.0001**	1:75
**B2, homo**	*05 neg	*02/*02	9	7 (0.78)	2 (0.22)	664 (50–8790)	**<0.0001**	**<0.0001**	1:52
**DQ2.5 homo**	*05/any	*02/*02	108	104 (0.96)	4 (0.04)	4422 (474–41281)	**<0.0001**	**<0.0001**	1:7

*p* values in boldface are significant

^a^X = any HLA-DQB1 allele different from *02 or *0302

^b^OR = Odds Ratio; 95%CI = 95% Confidence Intervals. Odds Ratios were calculated by binary logistic regression analysis adjusted for age, gender and *H*. *pylori* infection

^c^ Risk of CD is expressed as 1:N, where N is the number of individuals among which one case is present. Considering a disease prevalence of 1:184 in the general population [37], for each HLA-DQ category, N is calculated as a percentage of controls with that particular HLA-DQ status multiplied by 184 and divided by percentage of patients with the same DQ typing [10].

### *TNFA*, not *IFNG* and *TNFRSF1A* polymorphisms, are associated with CD

Table 3 reports the results of the studied polymorphisms of *TNFA* and *IFNG* genes. Considering each individual single nucleotide polymorphism (SNP), the minor allele frequency (MAF), the number and frequency of genotypes in cases and controls are shown. To ascertain whether minor alleles exert any effect on CD risk, we estimated the OR with 95% CI associated with dominant and recessive models (Table 3). With the exception of the *TNFA* -238G>A, all the studied *TNFA* SNPs were correlated with a diagnosis of CD, and all the rare alleles had a dominant effect on CD risk. The *IFNG*+874A>T polymorphism was not correlated with CD. Interestingly *H*. *pylori* infection was associated with *IFNG* +874A>T (dominant model: chi-square = 4.73, p = 0.030), not with the other polymorphisms.

**Table 3:** *TNFA*, *IFNG* and *TNFRSF1A* gene polymorphisms in CD.

Gene	dbSNP	MAF^a^	Genotypes	Cases ^b^	Controls ^c^	Dominant model		Recessive model	
				n (freq)	n (freq)	p-value (2df)	OR^d^(95%CI)	p-value (2df)	OR^d^(95%CI)
***TNFA***	-1031T>C	C (0.027)	C/C	9 (0.04)	22 (0.08)	**0.027**	0.65(0.44–0.95)	**0.042**	0.41 (0.17–0.97)
			T/C	73 (0.30)	97 (0.36)				
			T/T	162 (0.66)	148 (0.56)				
	-857C>T	T (0.153)	T/T	3 (0.01)	8 (0.03)	**<0.0001**	0.42 (0.27–0.65)	0.316	0.48 (0.12–2.01)
			C/T	45 (0.19)	89 (0.33)				
			C/C	196 (0.8)	170 (0.64)				
	-376G>A	A (0.047)	A/A	2 (0.01)	0 (-)	**0.023**	2.25 (1.12–4.51)	-	-
			G/A	28 (0.11)	16 (0.06)				
			G/G	214 (0.88)	251 (0.94)				
	-308G>A	A (0.216)	A/A	20 (0.08)	3 (0.01)	**<0.0001**	4.76 (3.12–7.26)	**0.001**	10.09 (2.71–37.55)
			G/A	117 (0.48)	58 (0.22)				
			G/G	107 (0.44)	206 (0.77)				
	-238G>A	A (0.068)	A/A	2 (0.01)	1 (0.01)	0.241	1.41 (0.80–2.46)	0.841	1.28 (0.11–14.67)
			G/A	34 (0.14)	29 (0.11)				
			G/G	208 (0.85)	237 (0.88)				
***IFNG***	+874A>T	T (0.458)	T/T	52 (0.21)	56 (0.21)	0.929	0.98 (0.65–1.48)	0.810	1.06 (0.67–1.68)
			A/T	116 (0.48)	136 (0.51)				
			A/A	76 (0.31)	75 (0.28)				
***TNFRSF1A***	c.625+10A>G	A (0.363)	G/G	17 (0.33)	26 (0.39)	0.739	1.16 (0.47–2.76)	0.061	0.21 (0.04–1.07)
			G/A	32 (0.63)	31 (0.47)				
			A/A	2 (0.04)	9 (0.14)				

*p* values in boldface are significant

^a^MAF = minor allele frequency

^b^Cases: n = 244; n = 51 for *TNFRSF1A*

^c^Controls: n = 267; n = 66 for *TNFRSF1A*

^d^OR = Odds Ratio; 95%CI = 95% Confidence Intervals. Odds Ratios were calculated by binary logistic regression analysis adjusted for age, gender and *H*. *pylori* infection.

In a first series of 16 CD and 14 controls, exons 2-3-4-6 and the flanking intronic DNA sequences of the *TNFRSF1A* gene were screened by DHPLC to identify the presence of DNA sequence variants (heteroduplex). No variants were found on considering exons 2-3-4 and the adjacent intronic regions. Since in 9 CD and in 4 controls DHPLC analysis revealed the presence of a variant within amplicons spanning exon 6 and adjacent intronic regions, these regions were analyzed by DHPLC in a further series of 35 cases and 52 controls. At DNA sequence analysis, all variant samples were found to carry intronic c.625+10A>G (rs1800693) polymorphism. In our series of patients, differently from what reported in the dbSNP database (http://www.ncbi.nlm.nih.gov/projects/SNP/snp_ref.cgi?rs=1800693) (last accessed: 30^th^ January 2015), the minor allele was the c.625+10G, not the c.625+10A. This *TNFRSF1A* polymorphism was not correlated with a diagnosis of CD (Table 3).

The findings made on ascertaining whether any of the studied *TNFΑ*, *IFNG* and *TNFRSF1A* polymorphisms were associated with the HLA-DQ haplotypes are shown in S2 Table. Three polymorphisms (-857C>T, -376G>A and -308G>A) of *TNFΑ* gene were associated with HLA-DQ haplotypes, whereas the remaining two *TNFA*(-1031C>T and -238G>A), *IFNG* +874A>T and *TNFRSF1A* c.625+10A>G polymorphisms were not. Multivariate binary logistic regression analyses were then made considering CD diagnosis as outcome variable, while HLA-DQ haplotypes, the carriage of *TNFA* and *IFNG* rare alleles in homozygosis (recessive effect) or in heterozygosis (dominant effect) were predictor variables (S3 Table). The reduction in the risk of CD by the *TNFA* -1031 A rare allele and the increase by the *TNFA*-308 Α rare allele occurred independent of HLA-DQ haplotype effects.

### *TNFA* haplotypes are HLA-independent risk factors for CD

The studied polymorphisms of the *TNFA*, all closely located in the gene promoter region, are carried as different haplotypes. We therefore determined the haplotypes resulting from the combination of *TNFA*-1031T>C,-857C>T,-376G>A and -308G>A polymorphisms, which were singly correlated with CD diagnosis. A total of six haplotypes with an overall frequency above 10^-5^ were found: their relative frequencies in CD cases and controls and the OR with 95% CI with respect to the most frequent haplotype (CCGG) are reported in S4 Table. For a clearer reading of subsequent results, the six haplotypes were coded as H1 to H6. *TNFA* haplotypes combinations were then estimated for any studied subject: a total of 17 haplotype combinations were inferred and are shown together with their frequencies in CD and controls in S5 Table (chi-square = 103, p<0.0001). The H1/H1 and H1/H2 combinations were rare among CD cases (<6%) and frequent among controls (>94%), while the inverse was observed for the H4/H5 and H5/H5 combinations (>80% among CD and <14% among controls). The rare H4/H4, H6/H6 and H3/H6 were exclusive to CD. The frequency of the remaining haplotype combinations ranged from 27 to 72% among CD and 31 to 73% among controls. On the basis of the frequency recorded among CD cases, haplotypes combinations were grouped as follows: group A (frequency among CD<10%); group B (10–40%); group C (40–60%); group D (60–80%); group E (>80%). *TNFA* haplotype combinations groups were correlated with the HLA-DQ haplotypes (chi-square = 190, p<0.0001). In particular, group A was strongly associated with HLA-DQ negativity, whereas group E strongly associated with HLA-DQ2.5 homozygotes (S1 Fig).

**Fig 1:**
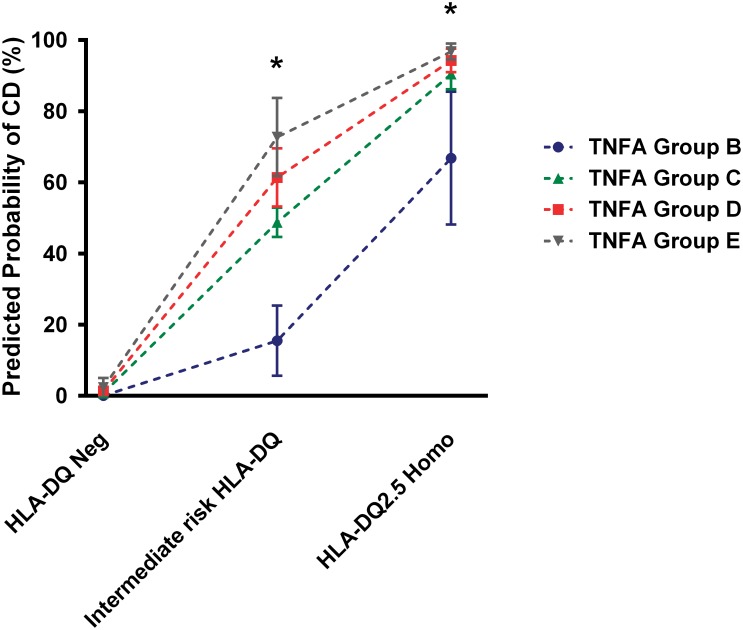
HLA-DQ and *TNFA* haplotype combinations predict CD risk. The marginal probability of CD was estimated on the basis of the multivariate binary logistic regression model reported in Table 4 adjusted for age, gender and *H*. *pylori* infection. Reference was the group of patients with negative HLA-DQ and *TNFA* group A haplotype combination. Dots and bars represent the estimated CD probability with their respective Bonferroni’s adjusted standard errors. *: p<0.05 for all groups with respect to *TNFA* Group A and p<0.05 for *TNFA* Group D with respect to *TNFA* Group B (pairwise comparisons of marginal linear predictions).

Table 4 shows the results obtained on ascertaining whether *TNFA* haplotype combinations exerted an independent effect on CD risk by performing multivariate binary logistic regression analysis adjusted for age, gender and *H*. *pylori* infection and considering CD diagnosis as an outcome variable, HLA-DQ haplotypes and *TNFΑ* haplotype combinations as predictors. *TNFΑ* haplotype combinations play an independent role in enhancing CD risk, although the ROC curve area obtained using the logistic regression model including as predictors HLA-DQ haplotypes, age, gender and *H*. *pylori* infection was not significantly different from the model that also included the *TNFA* haplotype combinations (p = 0.104) (S2 Fig). The multivariate binary logistic regression model presented in Table 4 was used to estimate the marginal probability of CD on the basis of HLA-DQ and *TNFA* haplotype combinations (Fig 1). The probability of CD was confirmed to be dependent on *TNFA* haplotype combinations mainly in the subset of patients carrying HLA-DQ intermediate risk haplotypes, which included HLA-DQ8, HLA-B2 hetero, HLA-DQ8/B1*02 pos, HLA-DQ2.5 hetero, HLA-DQ2/DQ8 and HLA-B2 homo.

**Table 4:** Binary logistic regression analysis.

	OR^a^	95%CI	p-value	Adjusted p-value^b^
**HLA-DQ haplotype:**				
**Neg**	Ref			
**Intermediate risk** ^c^ **haplotypes** ^c^	178	24–1317	**<0.0001**	**<0.0001**
**DQ2.5 homo**	2752	287–26387	**<0.0001**	**<0.0001**
***TNFA* haplotype: combinations**				
**Group A**	Ref			
**Group B**	5	1–28	**0.049**	0.490
**Group C**	11	2–65	**0.011**	0.110
**Group D**	13	2–70	**0.003**	**0.030**
**Group E**	13	2–104	**0.016**	0.160
**Constant**	0.0055	0.0004–0.07	**<0.0001**	-

Outcome variable: CD diagnosis; Predictor variables: HLA-DQ haplotypes, *TNFA* haplotype combinations.

*p* values in boldface are significant

^a^ OR = Odds Ratio; Odds Ratios were calculated by binary logistic regression analysis adjusted for age, gender and *H*. *pylori* infection

^b^Bonferroni’s adjusted p-values for multiple testing

^c^HLA-Q8, HLA-B2 hetero, HLA-DQ8/B1*02 pos, HLA-DQ2.5 hetero, HLA-DQ2/DQ8, HLA-B2 homo.

### *TNFA* haplotypes and CD histology

We verified whether HLA-DQ and *TNFA* genetics correlate with CD histology. Patients were classified on the basis of the presence or absence of total atrophy [38]. HLA-DQ haplotype was not significantly correlated with total atrophy, which was found in 64/104 (61.5%) HLA-DQ2.5 homozygotes and in 70/140 (50.0%) remaining CD patients (Odds ratio = 1.6, 95%CI = 0.92–2.77; p-value = 0.073). The risk of total atrophy was stratified for *TNFA* haplotype combinations groups A to E considering CD patients carrying a high (HLA-DQ2.5 homozygous) or intermediate risk HLA-DQ haplotypes. A significant decrease of the risk of total atrophy was found in the intermediate risk HLA-DQ group (OR = 0.72, 95% CI = 0.52–0.99, p = 0.048), but not in HLADQ2.5 homozygotes (OR = 0.99, 95% CI = 0.67–1.44, p = 0.941).

### Serum TNF-α increases in CD and associates with *TNFRSF1A*, not with *TNFA* genetics

**Fig 2:**
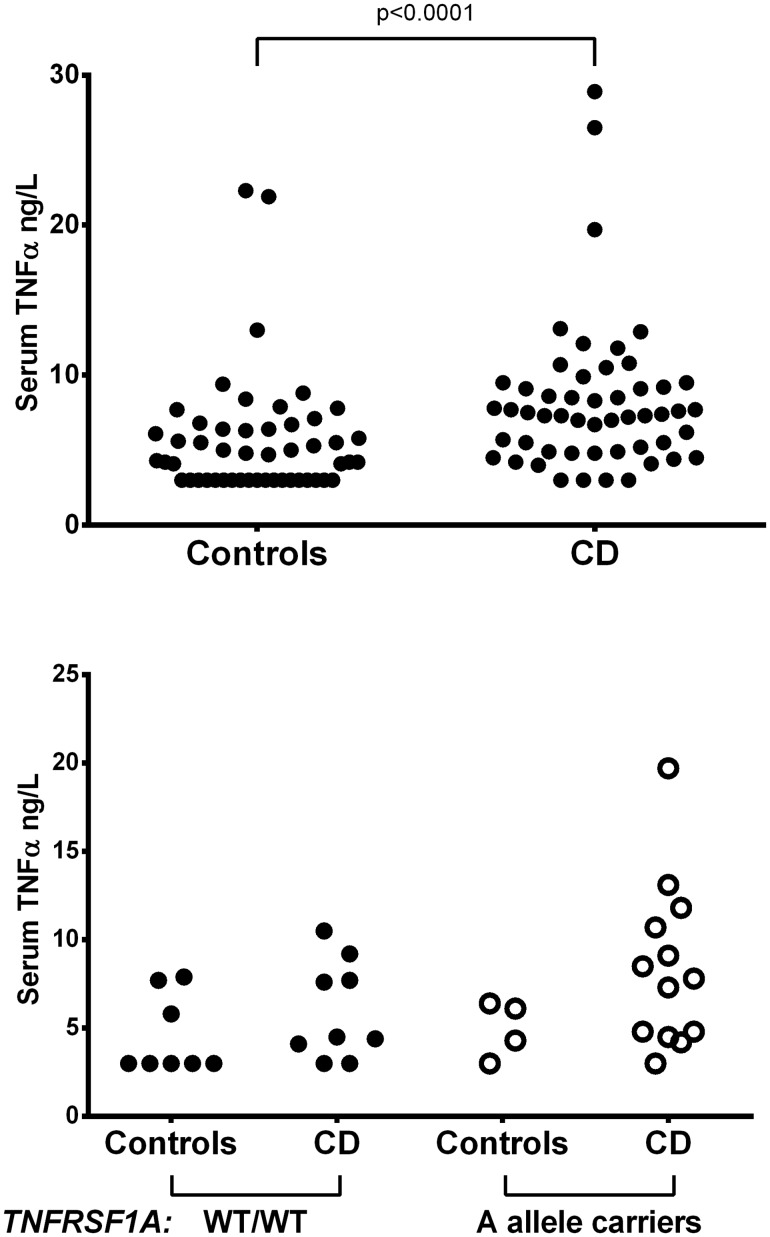
*TNFRSF1A* polymorphism affects serum TNF-α levels. Serum TNF-α in controls and CD patients (upper panel) and in patients subdivided according to the *TNFRSF1A*c.625+10A>G (rs1800693) polymorphism (lower panel).

We then verified whether serum TNF-α levels are influenced by CD presence and/or *TNFA*/*TNFRSF1Α* genetics. Serum TNF-α was measured in 52 cases and in 50 controls. Due to asymmetrical distribution (Shapiro-Wilk W test for normal data: p<0.0001), the variable was transformed to 1/square root. Although serum TNF-α values were significantly higher in CD patients than in controls (Student’s t test: t = 4.32, p<0.0001) (Fig 2, upper panel), they were not associated with any *TNFA* studied polymorphisms considered singly or as haplotypes (F = 0.98, p = 0.422). Interestingly, the circulating levels of TNF-α tended to be higher in CD patients carrying the *TNFRSF1A*A allele (one way Anova: F = 2.55, p = 0.0742; Fig 2, lower panel).

## Discussion

In the year 2012, the European Society for Pediatric Gastroenterology, Hepatology, and Nutrition formulated new guidelines for the diagnosis of CD in the pediatric age [39]. One algorithm was developed for symptomatic, and another for asymptomatic children and adolescents at an increased risk of developing the disease. In the former algorithm, positive findings for HLA-DQ2 or HLA-DQ8 are confirmatory for CD diagnosis in symptomatic children and adolescents with positive anti-tTG2 (10x normal) and EMA, while in the latter algorithm HLA-DQ2 or HLA-DQ8 testing is proposed as the initial action to identify negative patients who can be excluded from further studies. However, in patients positive at *HLA-DQA1* and *HLA-DQB1* typing, the CD risk depends on the resulting haplotype, spanning from very high (about 1:10 for HLA-DQ2.5) to low risk values (about 1:200 for B1*02/X), the latter being similar to that of the general population [10,37]. The findings made in the present study confirm the presence of a risk gradient for CD associated with the HLA-DQ haplotype resulting from *HLA-DQA1* and *HLA-DQB1* allele combinations: the HLA-DQ8 haplotype or the presence of HLA-DQB1*02 allele in single dose and not associated with HLA-DQA1*05 allele represent modest risk factors for CD, while the probability of developing the disease progressively increases as the number of HLA-DQB1*02 and HLA-DQA1*05 alleles increases, the highest risk (1:7) being found among HLA-DQ2.5 homozygotes. This high risk HLA-DQ haplotype was recently demonstrated to be the only factor that was significantly associated with the development of CD autoimmunity and overt CD in children who had at least one first-degree affected relative [40]. Since in our series of patients only few cases carried a single or double copy of the DQB1*02 allele associated with *DQA1* alleles different from DQA1*05 or carried the HLA-DQ8, in agreement with Lionetti et al. [40], our studied children were classified as having no HLA-DQ risk alleles, a high risk (HLA-DQ2.5 Homo) and an intermediate risk HLA-DQ haplotype, the latter including all together DQ8, B2 hetero, DQ8/B1*02 pos, DQ2.5 hetero, DQ2/DQ8 and B2 homo. From a pathophysiological viewpoint, the diversity in genetic susceptibility associated with different HLA-DQ haplotypes is probably due, at least in part, to the recognition of different gluten peptide repertoires affecting T cell activation [9]. From a practical clinical viewpoint this diversity raises the question of whether a different management should be proposed for asymptomatic patients carrying high risk or intermediate risk HLA-DQ haplotypes. More specifically, the identification of HLA-DQ independent genetic risk or protective factors for CD might improve our understanding of the disease and ameliorate risk stratification of patients. We focused our study on one HLA-associated gene, *TNFA* [26], and on two non-HLA-associated genes, *IFNG* and *TNRFSF1A* [32,33], whose protein products play an active role in the pathophysiology of CD. TNF-α and IFN-γ are known to play an active role in CD, but also in refractory CD [41], and sequence variations of the main TNF-α receptor, TNF-R1, caused by mutations of the encoding *TNRFSF1A* gene, may determine an altered cellular response to TNF-α stimulation [42].

*IFNG* +874A>T polymorphism was not associated with CD, but it was associated with *H*. *pylori*, thus further supporting the role of IFN-γ genetics in infectious diseases [32]. Four of the five *TNFA* promoter polymorphisms were found to be correlated with a diagnosis of CD: the rare alleles of the -1031T>C and -857C>T had a protective dominant effect, while the rare alleles of the -376G>A and -308G>A enhanced the risk of CD. The *TNFA*-308 A allele had an additional effect on CD risk, unlike the -376A allele; this difference is probably due to the fact that the latter minor allele frequency is very low (0.047) while the former is not (0.216). Since the *TNFA* gene is located in close proximity of the HLA class II region, we ascertained whether *TNFA* genotypes were associated with HLA-DQ haplotypes, and this occurred for the -857C>T, -376G>A and -308G>A, not for the -1031T>C, polymorphisms. To ascertain whether the *TNFA*-associated risk for CD is dependent or independent from its linkage with the *HLA-DQA1* and—*B1* genes, multivariate statistical analysis was performed, including as cofactors the potential confounders age, gender and *H*. *pylori* infection which was less frequently found among CD patients than in controls. The rare *TNFA*-1031 A and -308 A alleles were confirmed to be HLA-DQ independent CD-associated variants: carriers of the -1031 A allele in homozygosis have a reduced risk, while carriers of the -308 A allele have about a 3 fold increased risk of CD, this finding being in agreement with previously reported data in the literature [21,22,24].

To obtain further insight on the association between *TNFA* genetics and CD, our subsequent analysis of data was focused on *TNFA* haplotypes. Although any single polymorphism might influence gene transcription to different extents, in the presence of a string of SNPs located in close proximity with each other, their different combinations in haplotypes might reinforce or otherwise revert the effects on gene transcription of any single SNP. We considered the four *TNFA* SNPs significantly associated with CD (-1031T>C, -857C>T, -376G>A and -308G>A) and, using the Arlequin statistical software, estimated the haplotypes resulting from their combinations. Two of the six possible obtained haplotypes were more frequent among controls, the CCGG (H1) and TTGG (H2), three were more frequent among CD cases, the CCAG (H4), TCGA (H5) and CCGA (H6) haplotypes, while the frequency of the TCGG (H3) haplotype was similar in CD and controls. Since diploid cells carry pairs of haplotypes, we estimated the possible combinations resulting from H1–H6 haplotypesin our patients, and found 17 combinations. To reduce data dispersion five classes of haplotype combinations (groups A to D) were identified on the basis of their frequency among CD: very low (<10%), low (10–40%), intermediate (40–60%), high (60–80%) and very high (>80%). Haplotype combinations were confirmed to be correlated with CD diagnosis; although all *TNFA* haplotype combinations groups B to E increased CD risk with respect to group A, an HLA-DQ independent statistically significant effect was found for Group D with respect to group A. In the attempt to translate these findings in clinical practice, we verified whether *TNFA* haplotype combinations might improve risk estimation among the three HLA-DQ identified categories: HLA-DQ negative, HLA-DQ2.5 homozygotes and intermediate risk HLA-DQ. In the absence of HLA-DQ risk alleles, the *TNFA* haplotype did not modify the predicted CD risk. Vice versa in HLA-DQ high risk, but mainly in intermediate HLA-DQ risk subjects, carriage of any *TNFA* haplotype different from group A increased the predicted probability of disease and this increase appeared to be progressively higher in groups from B to E. Therefore *TNFA* genetic testing might be suggested as being of some help in improving CD risk assessment and in further studying patients. Interestingly, while HLA-DQ was not significantly associated with the histopathological lesion severity, in agreement with Ruiz-Ortiz et al. [43], *TNFA* haplotype A to E showed a negative trend of association with severity of lesions. In other words, the progressive increase in CD risk due to *TNFA* haplotypes is associated with a progressive less severe histopathology. If confirmed, this finding indicates that patients carrying high risk *TNFA* haplotypes are at risk of a less severe mucosal damage with a consequent uncertainty in the histological diagnosis [44].

In order to ascertain whether *TNFA* haplotypes influence TNF-α protein levels, we measured this cytokine in a series of case and control sera. Serum TNF-α values were higher in CD patients than in controls, supporting the role of TNF-α in CD, but they were not correlated with *TNFA* haplotypes. Although these findings argue against the hypothesis that *TNFA* haplotypes increase CD risk by enhancing TNF-α production, we cannot rule out that they affect the amount of TNF-α produced in the affected duodenal mucosa microenvironment.

One of the main TNF-α receptors mediating its the inflammatory effect is TNF-R1, encoded by the *TNFRSF1A* gene [45]. The R92Q polymorphism of this gene has been associated with an increased risk of TRAPS [46]. Due to the relevant impact of TNF-α on CD pathogenesis, we verified whether the *TNFRSF1A* gene had any variant correlated with CD. Only one polymorphism of this gene was identified in our series of cases and controls, the c.625+10A>G (rs1800693), which is reportedly a risk factor for multiple sclerosis [47]. The lack of any association between this polymorphism and CD allows us to rule out the hypothesis that it takes a part in causing duodenal atrophy upon exposure to gluten peptides. However, in CD patients this SNP was associated with slightly increased serum TNF-α levels. This might be due to an altered TNF-α/TNF-R1 balance consequent to the synthesis of a truncated TNF-R1 in patients carrying this polymorphism, as previously demonstrated by Ottoboniet al. [48].

In this study we have demonstrated that extended *TNFA* promoter haplotypes are HLA-DQ independent risk factors for CD and that their analysis might enhance the reliability in estimating the risk of CD especially in patients carrying an intermediate risk HLA-DQ haplotype. The strength of our principal findings rely upon the large number of patients with the same ethnicity entered the study. This renders our results transferrable within European populations, but require confirmation in populations that have a different LD structure, like Africans, Japanese or Chinese. The extended analysis of other genetic loci reportedly associated with CD by GWAS, which will be fast and cost-effective by the incoming high-throughput technology, represents a focus for future research.

## Supporting Information

S1 Fig *TNFA* haplotype combinations and HLA. Groups A to E definition in S5 Table.
(TIF)

S2 Fig ROC curves obtained using the logistic regression model for diagnosing CD. Predictors blu line: HLA-DQ haplotypes, age and gender; predictors red line: HLA-DQ haplotypes, *TNFA* haplotype combinations, age and gender.
(TIF)

S1 File Materials and Methods. (DOCX)

S1 Table Primers used for *TNFRSF1A* DHPLC and sequence analyses. (DOCX)

S2 Table Association between HLA and *TNFA*, *IFNG* and *TNFRSF1A* gene polymorphisms. (DOCX)

S3 Table Binary logistic regression analyses made considering CD diagnosis as outcome variable, while HLA-DQ haplotypes, the carriage of *TNFA* and *IFNG* rare alleles in homozygosis (recessive effect) or in heterozygosis (dominant effect) were predictor variables. (DOCX)

S4 Table Haplotypes resulting from the combination of *TNFA* -1031T>C, -857C>T, -376G>A and -308G>A polymorphisms (Arlequin). The OR with 95% CI were calculated with respect to the most frequent haplotype (TCGG)[36].
(DOCX)

S5 Table *TNFA* haplotype combinations. (DOCX)
