# Supplementary figures and images for: TNFA Haplotype Genetic Testing Improves HLA in Estimating the Risk of Celiac Disease in Children
Source: PLoS One. 2015 Apr 27;10(4):e0123244. doi: 10.1371/journal.pone.0123244 (PMC4411089; doi:10.1371/journal.pone.0123244)

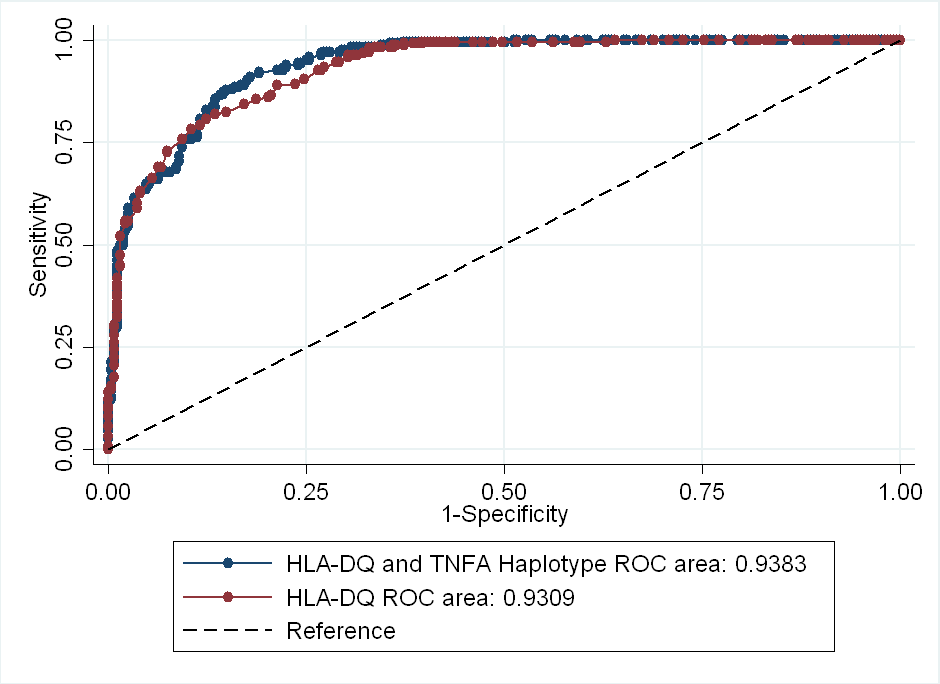

Supplement: S2 Fig — Predictors blu line: HLA-DQ haplotypes, age and gender; predictors red line: HLA-DQ haplotypes, TNFA haplotype combinations, age and gender. (TIF) [file pone.0123244.s002.tif]
