# Supplementary material for: TNFA Haplotype Genetic Testing Improves HLA in Estimating the Risk of Celiac Disease in Children
Source: PLoS One. 2015 Apr 27;10(4):e0123244. doi: 10.1371/journal.pone.0123244 (PMC4411089; doi:10.1371/journal.pone.0123244)
Supplement: S1 File — (DOCX) [file pone.0123244.s003.docx]

**S1 File. Materials and Methods**

**Serum tTG and TNF-α**

Fasting blood samples, collected immediately before endoscopy, were centrifuged at 3,000 g for 5 minutes to obtain sera, respective parts of which were used to determine IgA tTG (ELISA assay, QUANTA Lite IgAtTG, INOVA Diagnostics, San Diego, CA, USA). Serum TNF-α assay was performed using immunometric assay (IMMULITE-Siemens, Milano, Italy) in a series of 102 frozen (-80 °C) sera aliquots.

***TNFRSF1A* analysis**

***TNFRSF1A* Denaturing high-performance liquid chromatography (DHPLC; Wave 2100 Fragment Analysis, Transgenomic, Omaha, NE, USA)**

Exons 2, 3, 4 and 6 of *TNFRSF1Α* were PCR amplified using 120 ng of genomic DNA, 240 nM for each primer (Supplementary Materials and Methods Table), 200 μM of dNTPs, 1.5 mM of MgCl_2_ and 2.5 U of Taq polymerase (AmpliTaq Gold, Applied Biosystems) in a final volume of 50 μl. PCRs were run with an initial denaturation at 94 °C for 7 minutes, followed by 35 cycles at 94 °C for 30 seconds, 55 °C for 45 seconds and 72 °C for 45 seconds, with a final extension at 72 °C for 20 minutes. The amplification products were further processed with and without 1:1 dilution with the amplification products obtained from a control individual known to carry no variant alleles at the *TNFRSF1A* locus. 30 μL of undiluted and 1:1 diluted amplification products were denatured, reannealed and analyzed for heteroduplexes. The temperatures of DHPLC analyses for each analyzed exon are reported in Supplementary Materials and Methods Table.

***TNFRSF1A* gene sequencing (ABI PRISM 3130 Genetic Analyzer, Applied Biosystem)**

*TNFRSF1A* gene sequencing was performed using 2 μL of previously purified (ExoSAP, GE Healthcare, Fairfield, CT, USA) amplification product mixed with 300 nM of primer, and 4 μl of Big Dye terminator Mix (Applied Biosystem) in a final volume of 20 μL. Chromatograms were analyzed with Chromas Lite 2.01 software (Technelysium Pty Ltd., South Brisbane, QLD, Australia).
