# Supplementary material for: TNFA Haplotype Genetic Testing Improves HLA in Estimating the Risk of Celiac Disease in Children
Source: PLoS One. 2015 Apr 27;10(4):e0123244. doi: 10.1371/journal.pone.0123244 (PMC4411089; doi:10.1371/journal.pone.0123244)
Supplement: S1 Table — (DOCX) [file pone.0123244.s004.docx]

**S1 Table.** Primers used for *TNFRSF1A* DHPLC and sequence analyses. The temperatures used for DHPLC analyses are also reported.

| **Exon** | **F-primer** | **R-primer** | **DHPLC Temperature** |
| --- | --- | --- | --- |
| 2 | 5’TCCCTCTTTGATGGTGTCTCC3’ | 5’CAGACCTGAGGGCATTCACC3’ | 59.3 °C and 59.8 °C |
| 3 | 5’TTCCTTGTGTTCTCACCCGCAG3’ | 5’TCAAGACCCGCCTGACTCTC3’ | 62.7 °C and 63.2 °C |
| 4 | 5’AGGATGCAGGACTCATACCC3’ | 5’AAAGGAAGTGCCACCGCATGG3’ | 63.5 °C |
| 6 | 5’GTGTTCCTCCAATGGTAGGG3’ | 5’AAGCAGGTGTTGGTCAGAGG3’ | 60.5 °C and 61.0 °C |
