# Supplementary material for: TNFA Haplotype Genetic Testing Improves HLA in Estimating the Risk of Celiac Disease in Children
Source: PLoS One. 2015 Apr 27;10(4):e0123244. doi: 10.1371/journal.pone.0123244 (PMC4411089; doi:10.1371/journal.pone.0123244)
Supplement: S2 Table — (DOCX) [file pone.0123244.s005.docx]

**S2 Table. Association between HLA and *TNFA*, *IFNG* and *TNFRSF1A* gene polymorphisms.** Number and frequency (in brackets) are reported.

| **Gene** | **dbSNP** | **Genotype** | **HLA Neg (n=159)** | **HLA B2, Hetero (n=27)** | **HLA B2, Homo (n=9)** | **HLA DQ8 (n=24)** | **HLA DQ8/ B1*02 pos (n=8)** | **HLA DQ2/DQ8 (n=14)** | **HLA DQ2.5 Hetero (n=162)** | **HLA DQ2.5 Homo (n=108)** | **p-value** |
| --- | --- | --- | --- | --- | --- | --- | --- | --- | --- | --- | --- |
|  |  |  | **n (freq)** | **n (freq)** | **n (freq)** | **n (freq)** | **n (freq)** | **n (freq)** | **n (freq)** | **n (freq)** |  |
| ***TNFA*** | -1031T>C, rs1799964 | C/C | 11 (0.07) | 0 | 1 (0.11) | 3 (0.13) | 0 | 1 (0.07) | 8 (0.05) | 7 (0.06) | 0.150 |
|  |  | T/C | 63 (0.40) | 7 (0.26) | 1 (0.11) | 10 (0.42) | 5 (0.63) | 5 (0.36) | 53 (0.33) | 26 (0.24) |  |
|  |  | T/T | 85 (0.53) | 20 (0.74) | 7 (0.78) | 11 (0.46) | 3 (0.37) | 8 (0.57) | 101 (0.62) | 75 (0.70) |  |
|  | -857C>T, rs1799724 | C/C | 89 (0.56) | 18 (0.67) | 8 (0.89) | 20 (0.83) | 8 (1.00) | 10 (0.71) | 118 (0.73) | 95 (0.88) | **<0.0001** |
|  |  | C/T | 64 (0.40) | 9 (0.33) | 1 (0.11) | 3 (0.13) | 0 | 4 (0.29) | 40 (0.25) | 13 (0.12) |  |
|  |  | T/T | 6 (0.04) | 0 | 0 | 1 (0.04) | 0 | 0 | 4 (0.02) | 0 |  |
|  | -376G>A, rs1800750 | A/A | 0 | 0 | 0 | 0 | 0 | 0 | 0 | 2 (0.02) | **0.011** |
|  |  | G/A | 5 (0.03) | 1 (0.04) | 0 | 0 | 0 | 3 (0.21) | 19 (0.12) | 16 (0.15) |  |
|  |  | G/G | 154 (0.97) | 26 (0.96) | 9 (1.00) | 24 (1.00) | 8 (1.00) | 11 (0.79) | 143 (0.88) | 90 (0.83) |  |
|  | -308G>A, rs1800629 | A/A | 0 | 1 (0.04) | 1 (0.11) | 1 (0.04) | 0 - | 0 | 4 (0.03) | 16 (0.15) | **<0.0001** |
|  |  | G/A | 28 (0.18) | 3 (0.11) | 2 (0.22) | 4 (0.17) | 1 (0.12) | 5 (0.36) | 75 (0.46) | 57 (0.53) |  |
|  |  | G/G | 131 (0.82) | 23 (0.85) | 6 (0.67) | 19 (0.79) | 7 (0.88) | 9 (0.64) | 83 (0.51) | 35 (0.32) |  |
|  | -238G>A, rs361525 | A/A | 1 (0.01) | 0 | 0 | 0 | 0 | 0 | 0 | 2 (0.02) | 0.476 |
|  |  | G/A | 16 (0.10) | 2 (0.07) | 0 | 2 (0.08) | 0 | 3 (0.21) | 20 (0.12) | 20 (0.18) |  |
|  |  | G/G | 142 (0.89) | 25 (0.93) | 9 (1.00) | 22 (0.92) | 8 (1.00) | 11 (0.79) | 142 (0.88) | 86 (0.80) |  |
| ***IFNG*** | +874A>T, rs2430561 | A/A | 45 (0.28) | 10 (0.37) | 4 (0.45) | 5 (0.21) | 3 (0.38) | 5 (0.36) | 48 (0.30) | 31 (0.29) | 0.967 |
|  |  | A/T | 77 (0.49) | 12 (0.44) | 3 (0.33) | 14 (0.58) | 4 (0.50) | 5 (0.36) | 79 (0.49) | 58 (0.54) |  |
|  |  | T/T | 37 (0.23) | 5 (0.19) | 2 (0.22) | 5 (0.21) | 1 (0.12) | 4 (0.28) | 35 (0.21) | 19 (0.17) |  |
| ***TNFRSF1A* ^a^** | c.625+10A>G,  rs1800693 | G/G | 16 (0.47) | 0 | 0 | 1 (0.20) |  |  | 13 (0.32) | 13 (0.39) | 0.672 |
|  |  | A/G | 14 (0.41) | 3 (1.00) | 1 (1.00) | 3 (0.60) |  |  | 24 (0.58) | 18 (0.55) |  |
|  |  | A/A | 4 (0.12) | 0 | 0 | 1 (0.20) |  |  | 4 (0.10) | 2 (0.06) |  |

^a^ = The *TNRSF1A* c.625+10A>G polymorphism was typed in a subset of 117 patients (51 cases and 66 controls)
