# Supplementary material for: TNFA Haplotype Genetic Testing Improves HLA in Estimating the Risk of Celiac Disease in Children
Source: PLoS One. 2015 Apr 27;10(4):e0123244. doi: 10.1371/journal.pone.0123244 (PMC4411089; doi:10.1371/journal.pone.0123244)
Supplement: S3 Table — (DOCX) [file pone.0123244.s006.docx]

**S3 Table.** Binary logistic regression analyses made considering CD diagnosis as outcome variable, while HLA-DQ haplotypes classified as negative, DQ2.5 Homo or as intermediate risk haplotypes (DQ8, B2 hetero, DQ8/B1*02 pos, DQ2.5 Hetero, DQ2/DQ8, B2, Homo), the carriage of *TNFA* and *IFNG* rare alleles in homozygosity (recessive effect) or in heterozygosity (dominant effect). Odds Ratios (OR) were calculated by binary logistic regression analysis adjusted for age, gender and *H. pylori* infection.

| **VARIABLES** | | | **DOMINANT** | | | **RECESSIVE** | | |
| --- | --- | --- | --- | --- | --- | --- | --- | --- |
|  |  |  | **OR** | **95%CI** | **p-value** | **OR** | **95%CI** | **p-value** |
| **HLA-DQ haplotype Negative** | | | Ref. | - | **-** | Ref. | - | **-** |
| **HLA-DQ intermediate risk haplotypes** | | | 205 | 27-1530 | **<0.0001** | 237 | 32-1758 | **<0.0001** |
| **HLA-DQ haplotype DQ2.5 Homo** | | | 3672 | 377-35737 | **<0.0001** | 4894 | 510-46951 | **<0.0001** |
| ***TNFA*** | | **-1031T>C** | 0.94 | 0.48-1.84 | 0.864 | 0.13 | 0.03-0.54 | **0.005** |
|  |  | **-857C>T** | 1.20 | 0.61-2.36 | 0.601 | 0.98 | 0.14-6.64 | 0.983 |
|  |  | **-376G>A** | 2.87 | 0.37-22.06 | 0.468 | - | - | - |
|  |  | **-308G>A** | 2.99 | 1.60-5.60 | **0.001** | 1.55 | 0.32-7.60 | 0.588 |
|  |  | **-238G>A** | 0.50 | 0.08-3.24 | 0.465 | - | - | - |
| ***INFG*** | **+874A>T** | | 0.82 | 0.45-1.50 | 0.526 | 1.70 | 0.85-3.41 | 0.136 |
