# Supplementary material for: TNFA Haplotype Genetic Testing Improves HLA in Estimating the Risk of Celiac Disease in Children
Source: PLoS One. 2015 Apr 27;10(4):e0123244. doi: 10.1371/journal.pone.0123244 (PMC4411089; doi:10.1371/journal.pone.0123244)
Supplement: S4 Table — The OR with 95% CI were calculated with respect to the most frequent haplotype (TCGG)[36]. (DOCX) [file pone.0123244.s007.docx]

**S4 Table.** Haplotypes resulting from the combination of *TNFA* -1031T>C, -857C>T, -376G>A and -308G>A polymorphisms (haplologit package; Stata 13.1) [36]. The Odds Ratios (OR) with 95%CI were calculated with respect to the most frequent haplotype (TCGG) and were adjusted for age, gender and *H. pylori* infection. Haplotypes were progressively coded (H1 to H6) on the basis of progressive increase of Cases/Controls ratio.

| **HAPLOTYPE** | **Haplotye (H) code** | **FREQUENCY** | | **Cases/Controls Ratio** | **OR (95%CI)** | **p-value** |
| --- | --- | --- | --- | --- | --- | --- |
|  |  | **Cases** | **Controls** |  |  |  |
| **TCGG** | **H3** | 0.40 | 0.42 | 0.95 | Ref. | - |
| **CCGG** | **H1** | 0.11 | 0.23 | 0.48 | 0.52 (0.35-0.77) | **0.001** |
| **TTGG** | **H2** | 0.10 | 0.20 | 0.50 | 0.52 (0.35-0.78) | **0.002** |
| **CCAG** | **H4** | 0.07 | 0.03 | 2.30 | 2.01 (1.08-3.75) | **0.027** |
| **TCGA** | **H5** | 0.31 | 0.12 | 2.58 | 3.12 (2.14-4.53) | **<0.0001** |
| **CCGA** | **H6** | 0.01 | Less than 10^-5^ | - | 1.45 (0.09-23.14) | 0.793 |
