# Supplementary material for: TNFA Haplotype Genetic Testing Improves HLA in Estimating the Risk of Celiac Disease in Children
Source: PLoS One. 2015 Apr 27;10(4):e0123244. doi: 10.1371/journal.pone.0123244 (PMC4411089; doi:10.1371/journal.pone.0123244)
Supplement: S5 Table — (DOCX) [file pone.0123244.s008.docx]

**S5 Table.** *TNFA* haplotype combinations. Number and frequency (in brackets) of CD cases and controls are reported. Odds Ratios were calculated by binary logistic regression analysis adjusted for age, gender and *H. pylori* infection.

| **Classification (OR; 95% CI)** | ***TNFA* Haplotypes combinations** | | **Number** | **CD Nr. (frequency)** | **Controls Nr. (frequency)** | **CD/Controls ratio** |
| --- | --- | --- | --- | --- | --- | --- |
| **Group A** (Ref.) | H1/H1 | CCGG/CCGG | 17 | 1 (0.06) | 16 (0.94) | 0.06 |
|  | H1/H2 | CCGG/TTGG | 27 | 1 (0.04) | 26 (0.96) | 0.04 |
| **Group B (**11.2; 2.6-48.6) * | H1/H3 | CCGG/TCGG | 72 | 23 (0.32) | 49 (0.68) | 0.47 |
|  | H2/H2 | TTGG/TTGG | 11 | 3 (0.27) | 8 (0.73) | 0.40 |
|  | H2/H3 | TTGG/TCGG | 65 | 24 (0.37) | 41 (0.63) | 0.59 |
|  | H3/H3 | TCGG/TCGG | 83 | 32 (0.39) | 51 (0.61) | 0.64 |
| **Group C (**21.2; 4.5-100.3) * | H1/H4 | CCGG/CCAG | 11 | 5 (0.45) | 6 (0.55) | 0.82 |
|  | H2/H4 | TTGG/CCAG | 9 | 5 (0.56) | 4 (0.44) | 1.27 |
|  | H2/H5 | TTGG/TCGA | 33 | 15 (0.45) | 18 (0.55) | 0.82 |
| **Group D (**55.7; 12.5-247.8) * | H1/H5 | CCGG/TCGA | 35 | 23 (0.66) | 12 (0.34) | 1.94 |
|  | H3/H4 | TCGG/CCAG | 16 | 11 (0.69) | 5 (0.31) | 2.23 |
|  | H3/H5 | TCGG/TCGA | 96 | 69 (0.72) | 27 (0.28) | 2.57 |
| **Group E (**188.7; 30.6-1165.2) * | H4/H5 | CCAG/TCGA | 8 | 7 (0.88) | 1 (0.12) | 7.33 |
|  | H4/H4 | CCAG/CCAG | 2 | 2 (1.00) | 0 (-) | - |
|  | H5/H5 | TCGA/TCGA | 22 | 19 (0.86) | 3 (0.14) | 6.14 |
|  | H3/H6 | TCGG/CCGA | 3 | 3 (1.00) | 0 (-) | - |
|  | H6/H6 | CCGA/CCGA | 1 | 1 (1.00) | 0 (-) | - |

*: p<0.0001
